# Exercise Evaluation and Prescription

**DOI:** 10.3390/jfmk6010031

**Published:** 2021-03-23

**Authors:** Carl Foster, Cristina Cortis, Andrea Fusco

**Affiliations:** 1 Department of Exercise and Sport Science, University of Wisconsin-La Crosse, La Crosse, WI 54601, USA; cfoster@uwlax.edu; 2 Department of Human Sciences, Society and Health, University of Cassino and Lazio Meridionale, 03043 Cassino, Italy

Ever since the farm boy, Milo of Crotone, lifted a growing bullock every day, to become the strongest man in the world, and six-time champion of the ancient Olympic Games, we have known about the principle of progression of exercise training. Probably earlier, but certainly by the early 1950′s, Matti Karvonen in Finland [1] taught us that there was a minimal intensity of exercise training necessary to provoke a training response. Thus, we learned that prescription of training was based on an evaluation of the potential exerciser, in order to pick an appropriate relative training intensity [2]. Evaluative procedures that are highly individually specific are critical. By the mid 1970’s, several investigators demonstrated that various combinations of training frequency, intensity, type and training time (FITT) could produce predictable results in exercise capacity. This extensive body of knowledge is codified in documents like ACSM’s Guidelines for Exercise Testing and Prescription, now in its’ 10th edition [3]. 

Although training intensity and duration were originally based on the relative percent concept of Karvonen, more contemporary approaches have emphasized threshold-based prescription [4], and on simple psychophysiological approaches like rating of perceived exertion [5] and the Talk Test [6]. These latter approaches are somewhat evaluation independent in terms of prescribing the training load, but evaluation is still important in terms of assessing the outcomes of training.

Sometime in the intervening years, we learned about the concept of a “therapeutic window”, the dynamic space between the good effects and bad side-effects, such as myocardial infarction, that come from exercise training programs. We also learned about the interplay between the fitness that increases with training and the fatigue (often a precursor to injury) that comes with the same training program. This interplay is the nucleus of the training impulse (TRIMP) concept of Banister [7], which essentially underpins monitoring training programs. Within this space lies the business of exercise prescription. In athletes, operating outside the therapeutic window is likely to cause injuries that interfere with the goals of the training program. If they cause an athlete to miss important competitions, they can be quite meaningful, but are rarely permanent or life threatening. However, given the social and financial importance of contemporary high-level sport, missing such competitions simply because athletes ignored common sense advice is unreasonable. In the ever-increasing population of older exercisers, or of patients where exercise is part of a rehabilitation program, side effects can be more severe, often life threatening, although predictable and manageable [8]. Thus, understanding the parameters of the therapeutic window is critical to successful prescription of training programs.

This volume presents several papers, written from the perspective of optimizing training programs by better understanding the purpose and process of evaluating exercise capacity either in order to better prescribe exercise training or to better understand the outcome of exercise training programs. A total of 14 papers were published, including nine original articles, two viewpoints, two brief reports, and a review, focusing on healthy and sport population (soccer, off-road running, archery, dance, and pilot cadets) and diseases (type II diabetes, overweight, obese, breast cancer survivors, multiple sclerosis, and COVID-19 patients).

In order to carefully adjust training intensity and duration prescription, Foster et al. [9] suggested the utility of “translating” exercise test responses into the workload during exercise training. In particular, in sedentary individuals beginning an exercise program or in patients during rehabilitation, this approach may yield useful estimates of exercise intensity and contribute to both the safety and efficacy of exercise therapy. Accordingly, to implement physical interventions effectively, it is essential to provide an appropriate exercise and training prescription terminology. Therefore, Gronwald et al. [10] provided a new and clearer definition of the terms dose and response in the context of exercise and training prescription, suggesting that the dose of physical exercise and/or physical training should be operationalized by specific markers of internal load and modifying the exercise prescription by carefully adjusting the external load. 

Individualized and supervised training FITT prescription are particularly important for specific clinical populations and in particular situations such as the health-related consequences of COVID-19. In fact, Pippi et al. [11] with the C.U.R.I.A.Mo. Centre Experience showed the effectiveness and the importance of a supervised Nordic walk program to improve body weight control, body composition parameters, muscular flexibility and maximal oxygen uptake levels in obese adults with and without type 2 diabetes. Furthermore, Campa et al. [12] showed that a supervised high frequency resistance training program resulted in greater benefits for weight loss, cardiometabolic risk factors and handgrip strength than a training program with a session once a week in overweight and obese women. Additionally, the lack of significant associations between activity pacing and fatigue or physical activity found by Abonie et al. [13] suggests that people with multiple sclerosis which may benefit from targeted interventions to manage fatigue and optimize engagement in physical activity. Mascherini et al. [14] demonstrated that an exercise prescription program produces mid-term improvements in body composition, physical fitness and health-related quality of life of breast cancer survivors while adjuvant therapy slows down the effectiveness of an exercise program in the loss of fat mass. Individualization and personalization are also the key terms of the review proposed by Maugeri and Musumeci [15]. Accordingly, they provided a detailed review of the literature aiming to summarize updated evidence on the beneficial effects of adapted physical activity, based on personalized and tailor-made exercise, in preventing, treating, and counteracting the consequences of COVID-19. 

The dose–response relationship proposed by Gronwald et al. [10] depends on a multitude of factors, such as internal and external load, and influencing factors. Within the influencing factors, nutrition, hydration, anthropometrics, environment, sport specific circumstances and ability have been highlighted. Results from Magee et al. [16] demonstrated a continued need for sport nutrition education interventions to be part of regular team activities, recommended to help athletes understand their advanced dietary requirements, provide strategies to meet dietary recommendations and avoid low energy availability. Investigating the effect of dehydration on archery performance, subjective feelings and heart rate response, the study from Savvides et al. [17] reported that, despite the induced psychological and physiological strain, archery performance over 72 arrows was not affected by dehydration. Specific bioelectrical impedance vector analysis references for the start of the season period, through which the physical condition achieved after the preparation microcycle in soccer can be assessed, have been provided by Bongiovanni et al. [18]. Thanks to findings from Petri et al. [19], national and international federations will be able to perform regular body composition assessments using skinfold measurements in soccer referees. Rojas-Valverde et al. [20] showed that data related to impacts could better explain the cumulative mechanical kidney trauma during mountain running, suggesting technology to better understand how the number and magnitude of the g-forces involved in off-road running could potentially affect kidney function. Video observation and motor imagery training did not improve reaction time when compared to controls, but Sirico et al. [21] suggested it as a useful training strategy in individuals who need to simultaneously develop a fast response to different types of stimuli like pilot cadets. Finally, dance participation and experience proved to not influence balance and motor control in the sixth ballet position although resulting in better balance outcomes while standing in the first ballet position, suggesting identifying specific training adaptations and injury risk in varying foot positions [22]. 

Given the great success of the present Special Issue, we already launched a second edition, and we do hope to receive contributions focusing on the use of either laboratory or field evaluations to generate training advice in patients, healthy people, and athletes.
